# Epithelioid sarcoma with muscle metastasis detected by positron emission tomography

**DOI:** 10.1186/1477-7819-6-84

**Published:** 2008-08-15

**Authors:** Akio Sakamoto, Osamu Jono, Minako Hirahashi, Masafumi Oya, Yukihide Iwamoto, Ken Arai

**Affiliations:** 1 Department of Orthopaedic Surgery, Graduate School of Medical Sciences, Kyushu University, 3-1-1 Maidashi, Fukuoka, 812-8582, Japan; 2 Department of Orthopaedic Surgery, 3-83 Yoshio, Iizuka, Iizuka Hospital, 820-8505, Fukuoka, Japan; 3 Department of Pathology, 3-83 Yoshio, Iizuka, Iizuka Hospital, 820-8505, Fukuoka, Japan

## Abstract

**Background:**

Epithelioid sarcoma is an uncommon high-grade sarcoma, mostly involving the extremities.

**Case presentation:**

A 33-year-old man was referred to our institute with a diagnosis of Volkmann's contracture with the symptom of flexion contracture of the fingers associated with swelling in his left forearm. Magnetic resonance imaging (MRI) showed abnormal signal intensity, comprising iso-signal intensity on T1- and high-signal intensity on T2-weighted images surrounding the flexor tendons in the forearm. Diagnosis of epithelioid sarcoma was made by open biopsy, and amputation at the upper arm was then undertaken. [^18^F]-2-fluoro-2-deoxy-D-glucose-positron emission tomography (FDG-PET) detected multiple lesions with an increased uptake in the right neck, the bilateral upper arms and the right thigh, as well as in the left axillary lymph nodes, with maximum standardized uptake value (SUVmax) ranging from 2.0 to 5.5 g/ml. Magnetic resonance imaging confirmed that there was a lesion within the right thigh muscle which was suggestive of metastasis, even though the lesion was occult clinically.

**Conclusion:**

Increased uptake on FDG-PET might be representative of epithelioid sarcoma, and for this reason FDG-PET may be useful for detecting metastasis. Muscle metastasis is not well documented in epithelioid sarcoma. Accordingly, the frequency of muscle metastasis, including occult metastasis, needs to be further analyzed.

## Background

Epithelioid sarcoma was first described in 1970 [1]. Epithelioid sarcoma is an uncommon slow-growing malignant soft-tissue mass, usually found in the extremities, particularly in the hand and foot. The tumor is known to be associated with a high incidence of local recurrence and metastasis. The tumor is mostly prevalent in young adults aged between 20 and 40 years old [2]. The overall survival rates have been reported be 92.4%, 86.9% and 72.4% at 5, 10 and 15 years, respectively [3]. Epithelioid sarcoma has a diagnostic problem clinically, because its symptoms are sometimes similar to benign conditions, including inflammatory or granulomatous lesions [4].

[^18^F]-2-fluoro-2-deoxy-D-glucose-positron emission tomography (FDG-PET) has recently been used to assess various tumors. PET is evaluated using the standardized uptake value (SUV). An increased uptake of FDG-PET in cells reflects increased glucose metabolism as a result of various factors such as increased glucose transporters, high levels of hexokinase and a reduction in glucose-6-phosphatase [5,6]. FDG-PET has been reported to be useful for distinguishing malignant tumors from benign tumors in the case of lung tumors [7], head and neck tumors [8] and breast tumors [9]. As for bone and soft-tissue tumors, it has been reported that malignant tumors tend to have a higher SUV than benign tumors, with the cut-off point of SUV of 1.83 g/ml (sensitivity: 0.86, specificity: 0.42), 2.14 g/ml (sensitivity: 0.79, specificity: 0.52) and 3.23 g/ml (sensitivity: 0.57, specificity: 0.74) [10,11]. The same research group also reported that the cut-off point of SUV in bone lesions was 2.3 g/ml (sensitivity: 0.73), and in soft-tissue lesions it was 2.8 g/ml (sensitivity: 0.88) [10].

In this report, we present a case of epithelioid sarcoma with the symptom of Volkmann's contracture characterized by a claw-like deformity of the hand and fingers associated with contracture of the muscles in the forearm. Furthermore, FDG-PET detected occult metastasis to the muscle clinically, in addition to metastasis to the regional lymph nodes.

## Case presentation

**Figure 1:**
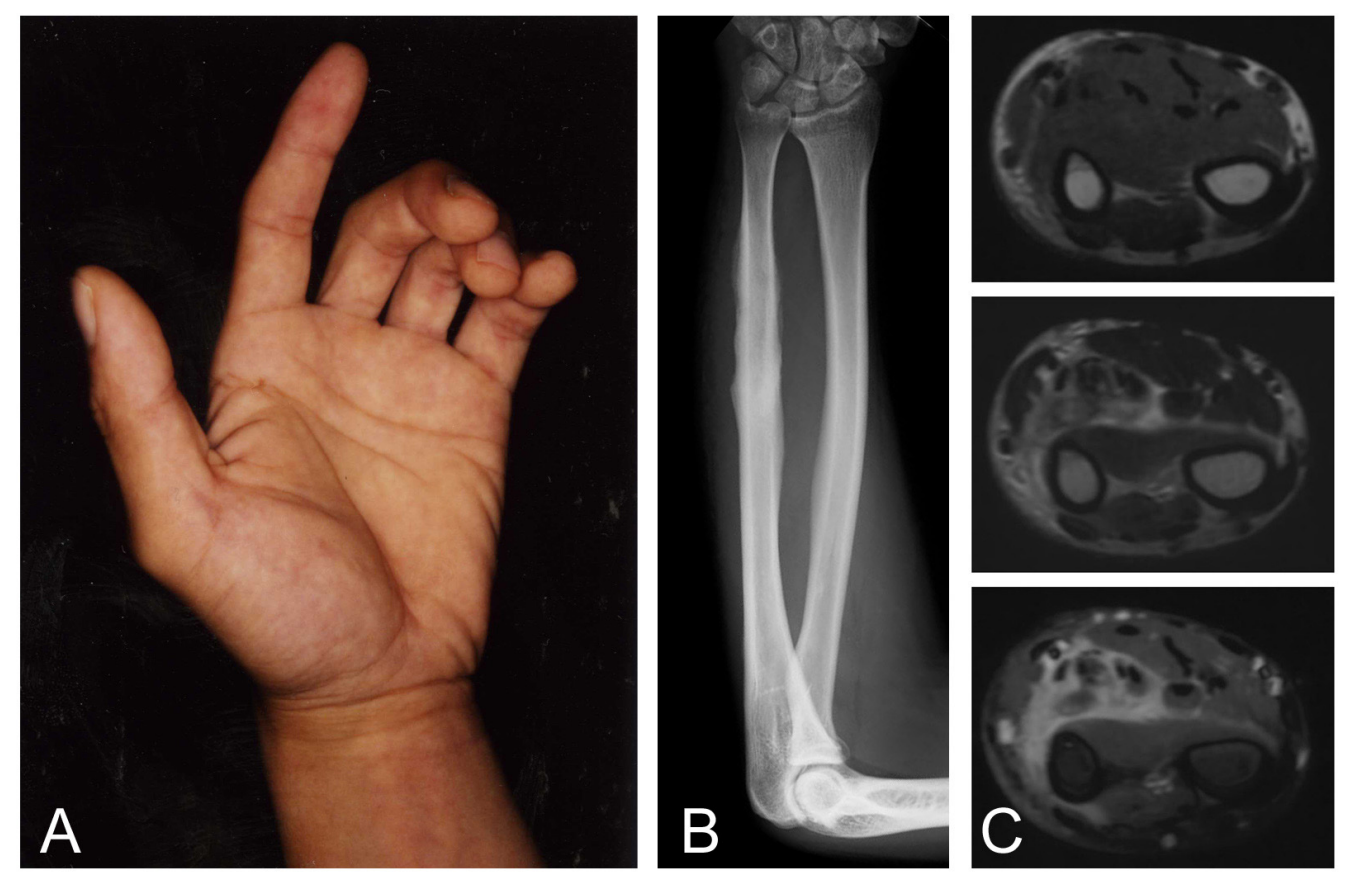
**Epithlioid sarcoma in the forearm.** Flexion contracture of the fingers can be seen (A). Plain radiograph shows irregular surface of the ulna (B). MRI of the forearm shows an abnormal lesion with iso-signal intensity on T1-weighted image (top) and high-signal intensity on T2-weighted image (middle) (C). Enhancement with gadolinium can be seen on T1-weighted fat-suppression image (bottom) (C).

A thirty-three-year-old man noticed extension disturbance of the left fingers, 7 months prior to the initial evaluation in our institute. He began to feel tension and pain in the forearm when he extended his fingers. He visited a local hospital 1 month after onset. A swelling in his left forearm appeared and this worsened gradually. The symptoms did not resolve, and the patient was then referred to our institute with a diagnosis of Volkmann's contracture (Figure 1A). Plain radiographs showed irregularity of the surface of the ulna, which was compatible with periostitis (Figure 1B). Magnetic resonance imaging (MRI) demonstrated a lesion surrounding the flexor tendons in the flexor compartments of the forearm with iso-signal intensity to the surrounding muscle tissue on T1-weighted images and heterogeneous high-signal intensity on T2-weighted images. The lesion was enhanced by gadolinium on T1-weighted images (Figure 1C). Based on these clinical symptoms and images, the cause of the Volkmann's contracture was explained as being due to chronic inflammation caused by repeated stress in the forearm, based on the facts that he was a carpenter and he used his upper extremities often. This was despite the fact that he was right-handed.

**Figure 2:**
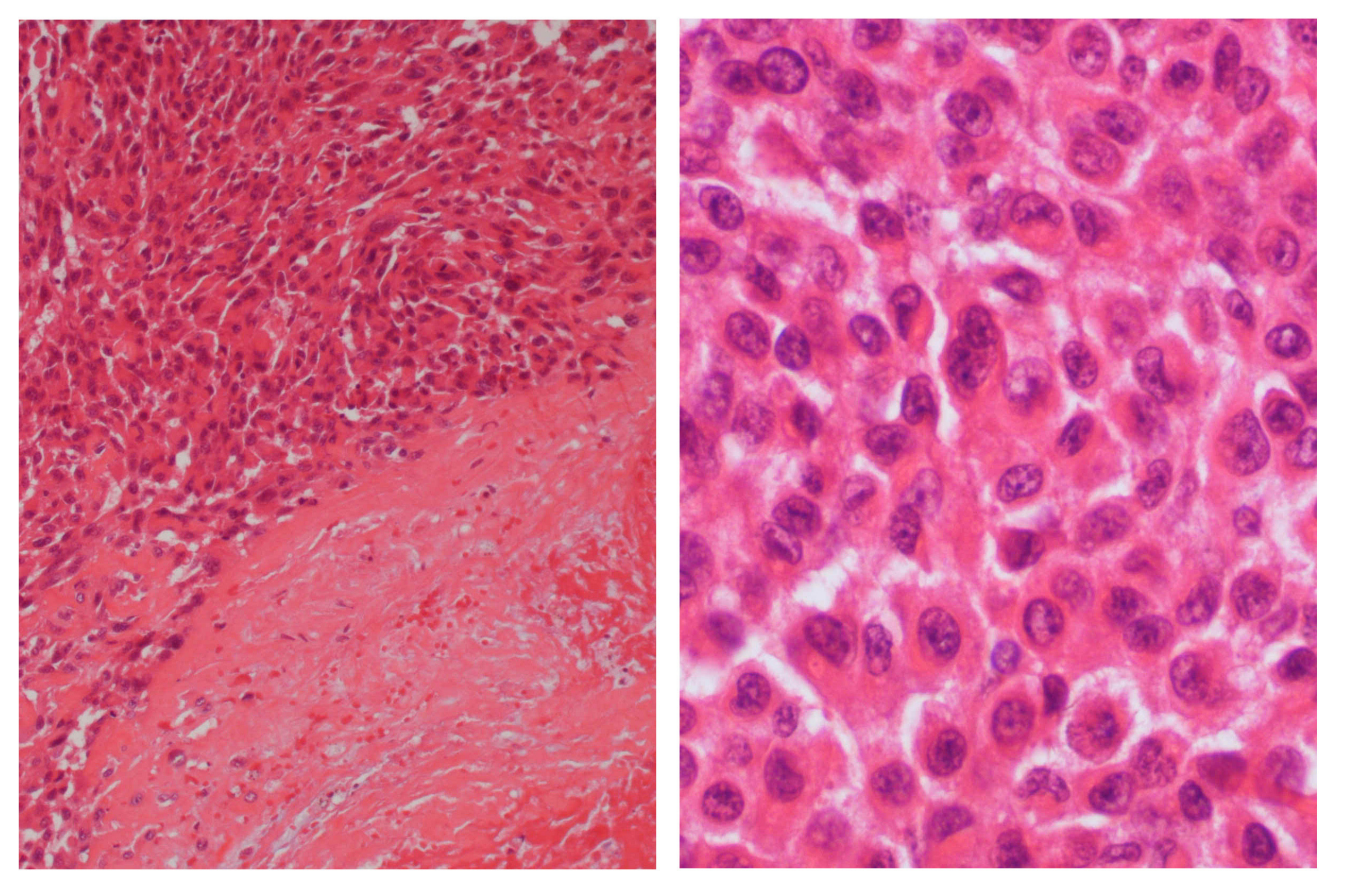
**Epithelioid sarcoma in the forearm shows atypical epithelioid cells in sheets associated with tumor necrosis (right portion) (A).** The neoplastic cells have nuclei and prominent nucleoli, with eosinophilic glassy cytoplasm (B). (Hematoxylin and Eosin original magnification; A, ×150; B, ×200).

At our institute, surgery was undertaken not only to release the contracture, but also to obtain a biopsy specimen to diagnose the cause of the contracture. The surgery findings showed that the tendons had adhered to each other with cicatricial-like tissue without any obvious mass lesion. Then, release of the adhered flexor tendons was undertaken. The cicatricial-like tissue was sampled for analysis, and histologically, it was found to be composed of rounded or polygonal epithelioid cells, arranged in sheets or a solid trabeculae pattern. Degeneration and necrosis were also observed (Figure 2A). The neoplastic cells had vesicular nuclei and prominent nucleoli, with characteristic eosinophilic glassy cytoplasm (Figure 2B). Immunohistochemically, the tumor cells were positive for an epithelial marker of EMA (epithelial membrane antigen) and cytokeratins (AE1/AE3, CAM5.2), but negative for S-100 protein, which is a Schwann-cell marker. These histological findings were typical of epithelioid sarcoma. However, epithelioid sarcoma needs to be differentiated from malignant soft-tissue tumors of epithelioid malignant peripheral nerve sheath tumor (MPNST) and malignant melanoma. Unlike epithelioid sarcoma, epithelioid MPNST tends to stain strongly for S-100 protein and virtually never expresses cytokeratins, whereas malignant melanoma virtually always expresses S-100 protein [4]. Some epithelioid sarcomas are also difficult to distinguish from epithelial tumor of ulcerating squamous cell carcinoma. However, epithelioid sarcoma lacks keratin pearls, as was true for the current case [4]. Taken together, a diagnosis of epithelioid sarcoma was made in the current case.

**Figure 3:**
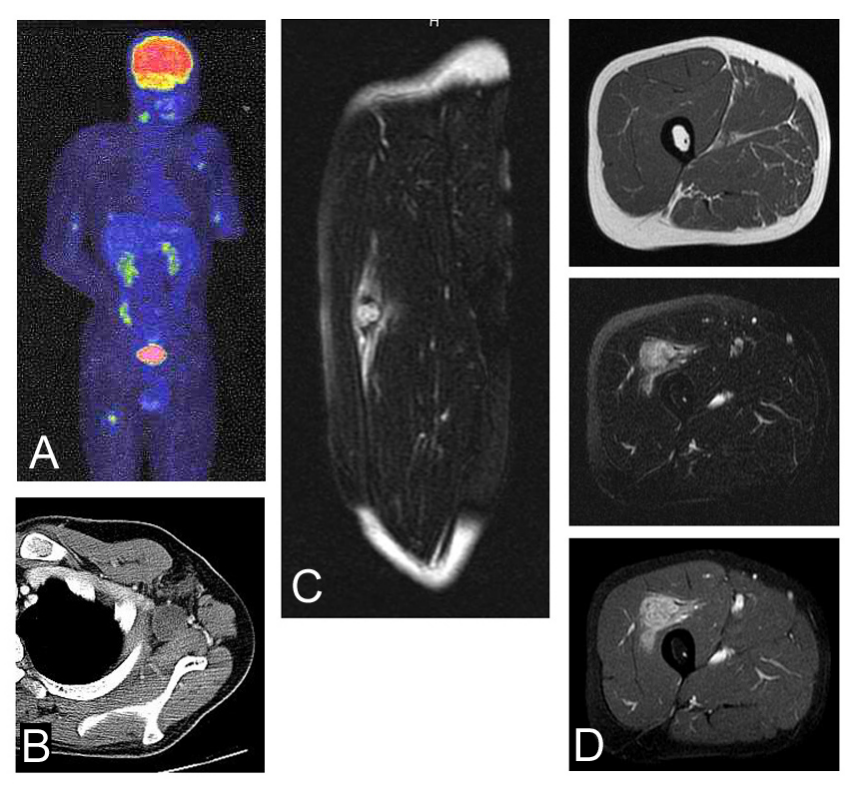
**Metastatic lesions of epithelioid sarcoma.** FDG-PET identifies lesions with an increased uptake in the right neck (SUVmax; 4.6), right upper arm (SUVmax; 4.1 g/ml), left upper arm (SUVmax; 4.2 g/ml), right thigh (SUVmax; 5.5 g/ml) left thigh (SUVmax; 2.0 g/ml), back (SUVmax; 3.6 g/ml), and lower back (SUVmax; 4.6 g/ml), as well as the left axilla (SUVmax; 3.9 g/ml) (arrows) (A). CT with contrast medium shows a swollen axillary lymph node (B). Sagittal MRI section of the right thigh shows a nodular lesion associated with prominent longitudinal abnormal signal intensity on T2-weighted image (C). The nodular lesion shows iso-intensity on T1-weighted fat-suppression image (top), and heterogeneous high-intensity on T2-weighted image (middle) (D). Enhancement with gadolinium can be seen on T1-weighted fat-suppression image (bottom) (D).

CT showed no evidence of pulmonary metastasis, but it showed mild swelling of the axillary lymph nodes, which could have been possible metastasis, or simply non-specific swelling due to the biopsy procedure. Amputation above the elbow was undertaken. Three months after the amputation, bone scintigraphy showed no evidence of abnormal findings suggestive of metastasis to the bone (data not shown). However, CT showed increased size of the axillary lymph nodes, suggesting that these lymph nodes were actually metastasized (Figure 3B). For further examination, FDG-PET was undertaken, and it detected multiple lesions with an increased uptake in the right neck (SUVmax; 4.6 g/ml), right upper arm (SUVmax; 4.1 g/ml), left upper arm (SUVmax; 4.2 g/ml), right thigh (SUVmax; 5.5 g/ml) left thigh (SUVmax; 2.0 g/ml), back (SUVmax; 3.6 g/ml), and lower back (SUVmax; 4.6 g/ml), as well as the left axilla (SUVmax; 3.9 g/ml) (Figure 3A). As for the right neck lesion, ultrasonography and CT failed to detect swelling of the lymph node just after FDG-PET examination, although the swollen lymph lesion was confirmed physically 3 months later (data not shown). The right thigh lesion with an increased uptake on FDG-PET was not palpable, and had no tenderness on physical examination. However, MRI demonstrated a nodular metastatic lesion measuring 2 × 2.5 cm which was located within the thigh muscle with iso-signal intensity to the muscle tissue on T1 images and heterogeneous high-signal intensity on T2-weighted images. Gadolinium enhancement on T1-weighted images was seen in the lesion. The surrounding reactive lesions were seen mainly longitudinally (Figure 3C). The thigh lesion was still not palpable 3 months after the FDG-PET examination.

## Discussion

A diagnosis of epithelioid sarcoma is challenging clinically, because epithelioid sarcoma is likely to be confused with a variety of benign and malignant conditions [4]. Due to this diagnostic difficulty, it has been reported that the median interval between observing the initial symptoms, making a diagnosis, and starting treatment is 3.5 months, ranging between 1 and 36 months [2]. In the current study, 7 months passed before a biopsy was undertaken for diagnosis, because the initial clinical diagnosis had been benign inflammatory lesion resulting in Volkmann's contracture. Furthermore, MRI failed to detect any obvious space-occupying lesion, and it was less suggestive of a solid tumor. Generally, in epithelioid sarcoma, necrosis within a neoplasm is a common finding. When the tumor spreads within a fascia or aponeurosis, it forms festoon-like or garland-line bands punctuated by areas of necrosis [4]. The spread pattern of the epithelioid sarcoma seems to have caused the clinical symptoms of Volkmann's contracture in the current case.

SUV in FDG-PET of malignant bone and soft-tissue lesions has been reported to be higher than that of benign bone and soft-tissue lesions, with the cut-off point ranging from 1.83 to 3.23 [10,11]. In another report, the cut-off point of SUV in FDG-PET in soft-tissue lesions has been reported to be 2.8 g/ml [10]. In a previous study, FDG-PET was reported to be useful for verifying adjacent bone marrow infiltration in a case of epithelioid sarcoma, in which there are 2 foci in the right gluteus (SUVmax; 4.0–6.1 g/ml) and sacrococcygeal (SUVmax; 7.0–7.5 g/ml) regions [12]. In the current case, the SUVmax of FDG-PET in the multiple metastatic lesions ranged from 2.0 to 5.5 g/ml. Taken together with the current case and the reported case, it might be possible that an increased uptake in FDG-PET is characteristic of epithelioid sarcoma.

In the current study, without FDG-PET, only the metastasis to the lymph nodes could be detected by CT, because metastasis to the muscle was occult clinically, even 3 months after the FDG-PET examination. The metastatic rate of epithelioid sarcoma has been reported to be 45%, and the most common sites of metastasis are the lung (51%), regional lymph nodes (34%), the scalp (22%) and bone (13%), while metastasis to the soft-tissue, including muscle, is thought to be less common [4]. However, taking into consideration the clinically occult muscle metastasis in the current case, it is possible that clinically occult metastasis, such as to the muscle, is much more common than has been thought. There has been a report that the presence of lymph node metastases is not a significantly unfavorable factor [2], in contrast to other studies with conflicting results [4,13-18]. It has also been suggested that lymph node metastases may be the first symptom of widely disseminated disease rather than a purely regional process [16].

## Conclusion

In conclusion, we have reported a case of epithelioid sarcoma with the symptom of Volkmann's contracture. The current case should act as a reminder that Volkmann's contracture can be a symptom of epithelioid sarcoma in the forearm, and this reminder should help avoid a delay in the commencement of treatment. Multiple metastases to the lymph nodes and muscle had an increased uptake in FDG-PET, and the increased uptake may be representative of epithelioid sarcoma. Metastasis to the muscle tissue has not been well described so far. Further reports are necessary for the precise frequency of muscle metastasis to be ascertained. In that case, FDG-PET might be useful for detecting such metastasis.

## Competing interests

The authors declare that they have no competing interests.

## Authors' contributions

AS drafted the manuscript. MH and MO are pathologists who helped with the discussion. AS, OJ and KA are surgeons who carried out the operation. YI is the Professor of the Department of Orthopaedic Surgery of Kyushu University who approves all relevant manuscripts. All authors read and approved the final manuscript.
